# Cellulose degradation: a therapeutic strategy in the improved treatment of *Acanthamoeba* infections

**DOI:** 10.1186/s13071-015-0642-7

**Published:** 2015-01-14

**Authors:** Sahreena Lakhundi, Ruqaiyyah Siddiqui, Naveed Ahmed Khan

**Affiliations:** Department of Biological and Biomedical Sciences, Aga Khan University, Stadium Road, Karachi, Pakistan

**Keywords:** *Acanthamoeba*, Cyst formation, Cyst wall, Cellulose

## Abstract

*Acanthamoeba* is an opportunistic free-living amoeba that can cause blinding keratitis and fatal brain infection. Early diagnosis, followed by aggressive treatment is a pre-requisite in the successful treatment but even then the prognosis remains poor. A major drawback during the course of treatment is the ability of the amoeba to enclose itself within a shell (a process known as encystment), making it resistant to chemotherapeutic agents. As the cyst wall is partly made of cellulose, thus cellulose degradation offers a potential therapeutic strategy in the effective targeting of trophozoite encased within the cyst walls. Here, we present a comprehensive report on the structure of cellulose and cellulases, as well as known cellulose degradation mechanisms with an eye to target the *Acanthamoeba* cyst wall. The disruption of the cyst wall will make amoeba (concealed within) susceptible to chemotherapeutic agents, and at the very least inhibition of the excystment process will impede infection recurrence, as we bring these promising drug targets into focus so that they can be explored to their fullest.

## Review

Protists are the largest, unicellular, non-photosynthetic eukaryotes that lack cell walls and include pathogens of both animals and humans. Pathogenic and opportunistic free living protist such as *Acanthamoeba* spp., *Balamuthia mandrillaris* and *Naegleria fowleri* occur worldwide and propagate independently in the environment. However upon accidental encounter with humans, they can produce disease [1-3]. *Acanthamoeba* species are now becoming increasingly important as human pathogens causing serious and life threatening infections. They are well known to cause blinding keratitis as well as granulomatous amoebic encephalitis [4]. The most distressing aspect of *Acanthamoeba* infections is their recurrence owing to the failure of therapy, attributed to the ability of the parasite to transform into a resistant cyst form [1-3]. *Acanthamoeba* undergoes two stages during its life cycle: a metabolically active “trophozoite” stage and a “cyst” stage with minimal metabolic activity. The trophozoite aggressively feeds on human cells as well as grazes upon bacteria, yeast and/or small organic particles and multiplies enthusiastically. Exposure to harsh environmental conditions i.e., in the face of famine, extremes of pH, temperature, osmolarity and the presence of antimicrobial drugs, results in cellular differentiation into a resistant cyst form [5]. During encystment, the trophozoite enfolds itself into a circular structure surrounded by a cyst wall with minimal metabolic activity. The transformation of the trophozoite from a metabolically active form to an inactive form can impede successful treatment. When encysted, the trophozoite encloses itself within a shell that makes it resistant to physiological concentrations of a number of physical, chemical and radiological conditions and chemotherapeutic agents [6,7]. It is envisaged that degradation of the shell (cyst wall) will allow effective killing of trophozoite within. The cyst wall consists of an ectocyst (laminar, fibrous outer layer) and an endocyst (fine, fibriler inner layer) [5,8,9]. Both layers are separated by space except at opercula in the centre of ostioles which during excystation serves as the exit point for trophozoite. The mature cyst therefore consists of a double-walled structure with the wall serving as a protective barrier to facilitate parasite survival of hostile conditions. As the cyst wall offers resistance to chemotherapeutic agents, it may leave amoeba viable to re-establish infection following antimicrobial chemotherapy [6]. Cyst walls remain intact even with the treatment of SDS plus boiling, EDTA, enzymes (including papain, DNase, RNase, amyloglucosidase, proteinase K) and DTT [10]. Resilient nature of cyst walls suggest that they are composed, at least in part, of carbohydrates possibly polysaccharides. This is in agreement with the fact that the carbohydrate analysis of cysts walls of *A. castellanii* using GC/MS showed that they contain 44.4% glucose [10]. The linkage analysis confirmed the presence of 4-linked glucopyranose (22.2%) in the cyst walls of *A. castellanii* which is suggestive of cellulose and is consistent with previous findings [11,12]. Dudley et. al., [13] observed the inhibition of *Acanthamoeba* encystment in the presence of 2,6-dichlorobenzonitrile, an inhibitor of cellulose synthesis. Furthermore, the enzymes for the synthesis and breakdown of cellulose have been identified in *A. castellanii*, indicating the presence of cellulose in the cyst walls of this organism [14]. Hence the sturdy nature of *Acanthamoeba* cysts is attributed, in part, to cellulose and suggests that cellulose could serve as a potential target. Previous work has described cellulose synthesis in *Acanthamoeba* [5,12,14-16]. In agreement with recent studies [17,18], here it is proposed that cellulose degradation offers a potential therapeutic strategy in effective targeting of trophozoite encased within the cyst walls. Here, we present a comprehensive report on the structure of cellulose and cellulases, as well as known cellulose degradation mechanisms with an eye to target *Acanthamoeba* cyst wall. It is envisaged that the disruption of cyst wall will make amoeba (concealed within) susceptible to chemotherapeutic agents, and at the very least inhibition of excystment process would impede infection recurrence and undoubtedly be of potential value in therapy.

### What is cellulose?

**Figure 1:**
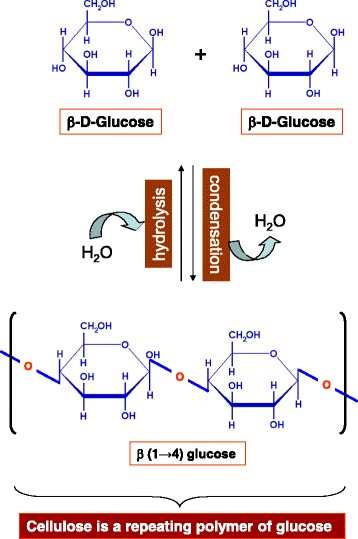
**Beta-D-glucose is the basic subunit of cellulose, which is an important component of the cyst wall of** ***Acanthamoeba*** **.**

Cellulose is the most abundant biopolymer on earth, a product of solar energy due to its origin from photosynthetic process. It is a major component of plant biomass and also produced in considerable amounts by green algae (*Valonia* and *Micrasterias*), slime mold *Dictyostelium*, bacteria (*Acetobacter xylinum*), sea animals (*Halocynthia*) and other animals such as tunicates etc. [19,20]. An exceptional feature of cellulose which is also relatively unusual in the polysaccharide world is its crystalline structure [20,21]. Cellulose is basically a linear polymer of β-1,4 linked glucose units (Figure 1), which are assembled at the site of its synthesis requiring the action of cellulose synthase and UDP. The β-1,4 linked *D*-glucose units are arranged in alternate orientation with respect to one another so that the repeating unit is cellobiose rather than glucose. Degree of polymerization in a cellulose molecule varies from 100 to 14,000 residues depending on the source of cellulose [22]. Unlike starch, cellulose is a straight chain polymer with no coiling and rod-like conformation that provokes spontaneous crystallization of the molecule [19]. Approximately 30 individual molecules of cellulose are assembled into larger units called elementary fibrils (protofibrils), which in turn are packed into larger units called microfibrils [20,21]. The chains in the microfibrils are held together by hydrogen bonds giving them a high tensile strength. It is this inter- and intra-chain hydrogen bonding between multiple parallel layers of cellulose that results in the formation of tightly packed microfibrils. The microfibrils then associate into crystalline cellulose fibers [22,23].

Cellulose, although it is said to have a crystalline structure, in nature these fibers are not purely crystalline. In the physical world, cellulose fibers range from purely crystalline to purely amorphous forms [20,24]. Depending on the origin, the degree of cellulose crystallinity can vary from 0% of that of amorphous, acid swollen cellulose, to 70% of that from cotton, to nearly 100% of that from *Valonia macrophysa* [25,26]. The organization of individual microfibrils in crystalline cellulose is such that the component molecules are packed tightly enough to prevent penetration by enzymes. In addition, cellulose also contain various irregularities such as twists or voids, surface micropores, large pits and capillaries etc., increasing the total surface area much larger than that of an ideally smooth fiber of the same dimensions [20,27-29]. Hence the net effect of this heterogeneity is that the fibers are partially hydrated when in aqueous medium, allowing the invasion of comparatively larger molecules like cellulases. Overall, cellulose imparts high tensile strength to the wall it is contained in, serving as a structural component. It is an extracellular polysaccharide and is a part of the cell wall in plants, algae, bacteria, slime mold *Dictyostelium* and other protists such as *Acanthamoeba* cyst wall [30-33].

### What are cellulases?

Cellulose degradation is carried out by the enzymes called “cellulases”, responsible for the hydrolysis of β-1,4-linkages present in cellulose [34,35]. Although chemically homogenous, cellulose exists in crystalline and amorphous topologies and no single enzyme is able to hydrolyze cellulose. Its insoluble, crystalline and heterogeneous nature makes it a resilient and challenging substrate for enzymatic hydrolysis [19]. Given the structure of cellulose, it practically makes it impossible for the enzyme to clasp cellulose into its substrate site and hence for a single enzyme to hydrolyze cellulose. This, together with its association with other polymers, makes cellulose containing material withstand harsh conditions making it hardy and resistant to degradation, hence its role as a structural and protective barrier. Cellulose, is therefore only hydrolyzed by a variety of simultaneously acting enzymes interacting with each other to bring complete hydrolysis. Consequently, true cellulolytic organisms produce a multiple-enzyme system [36-38]. These multiple-enzyme systems act in synergy to bring effective hydrolysis of cellulose. At least three different types of enzyme activities are required for complete hydrolysis of this polymeric substrate into its monomeric unit [20,39,40]:

- Endoglucanase activity
- Exoglucanase activity (also called cellodextrinase or cellobiohydrolase)
- β – glucosidases activity

**Figure 2:**
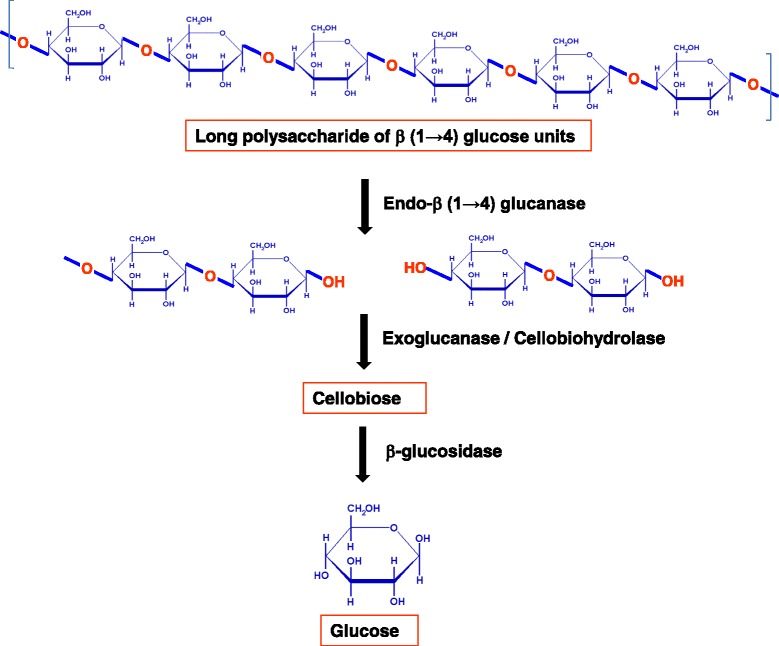
**Classes of enzymes involved in cellulose breakdown.** Endoglucanase produce random cuts at an internal position within cellulose fiber releasing cellooligosaccharides. Exoglucanases/cellobiohydrolase act on chain ends releasing cellobiose which are then acted up on by β-glucosidase to release glucose (adopted from Xie *et. al.,* 2007).

Only by the cooperation of these activities, enzymes are able to disrupt the structure at the solid–liquid interface making the individual fiber available for hydrolysis. Endoglucanases produce random internal cuts within the amorphous region in the cellulose molecule, yielding cello-oligosaccharides of various lengths and thereby generating new chain ends [20,40]. Exoglucanases act progressively on the reducing and/or non-reducing ends producing either glucose, cellobiose and/or cellooligosaccharides. These soluble cellodextrins and cellobiose are then hydrolyzed by β-glucosidases to glucose (Figure 2). Endoglucanases have an open active site, as they are able to bind to the interior of the long cellulose fibers [40]. This is in contrast to exocellulase, which have their active site in a tunnel and hence is consistent with their processive nature resulting in sequential release of cellobiose from the end of cellulose chain. The three types of enzymes act in a coordinated manner to hydrolyze cellulose. The amorphous regions within the cellulose fibers are first attacked by endoglucanase, creating sites for exoglucanases to proceed into the crystalline regions of the fiber [41]. They also tend to act on microcrystalline cellulose, to apparently peel the cellulose chains off its microcrystalline structure. Lastly, β-glucosidases split cellobiose to glucose preventing the build-up of cellobiose which inhibits cellobiohydrolases. Cellulolytic activity of cellulases, not only differ in the way they act on cellulose but also in the way they bind to the crystalline surface of their insoluble substrate. In fact all enzymes that act on insoluble substrates contain two binding sites: the “active site” which is usually contained in the catalytic domain of the enzyme and the “substrate binding site” which is the part of a separately folded and functionally distinct carbohydrate/cellulose binding domain [19,35,42-44]. The two domains are separated by linker peptide which acts as a flexible arm connecting the two parts together. Hence the structure of most cellulases includes a cellulose binding domain (CBD) and a catalytic domain (CD) as described below.

#### Cellulose binding domain (CBD)

**Figure 3:**
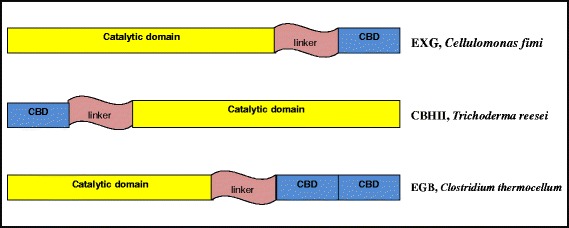
**Schematic representation of multi-domain organization shown by non-complex cellulases.** Cellulose binding domain exist as single, double or triple domain and could be located on either *C*- or *N* terminal of the protein. They are connected to catalytic domain via a linker sequence rich in proline and threonine. CBD is carbohydrate binding domain, EXG is exoglucanase, CBHII is cellobiohydrolase II, EGB is endoglucanase B.

In the late 1940s, Reese et al. [45] proposed that the initial stage of conversion of crystalline cellulose involves the action of an unknown non-hydrolytic component termed C_1_ [now known as Carbohydrate Binding Domain (CBD)]. This C_1_ system makes the substrate more accessible to the hydrolytic component C_x_ [now known as Catalytic Domain(CD)], by destabilizing the structure of cellulose [45,46]. Hence a CBD is defined as an adjoining amino acid sequence within a carbohydrate active enzyme with a distinct fold having carbohydrate binding activity. To date, more than 300 CBD sequences in more than 50 different species have been identified that have been classified into 64 different families, based on their amino acid sequences, structure and binding specificity [44]. Not all CBDs bind cellulose. Many families contain CBDs that bind to other carbohydrate polymers like chitin, xylan, starch etc. Those CBDs may be specific for one polymer or may have the ability to bind to several different polymers. Hence the presence of CBDs is a general property of carbohydrate acting enzyme [34,47]. The CBD exists as a single, double or triple domain in a protein and contains 30 – 200 amino acids. The location of CBD in a polypeptide chain could be both, *C*- or *N*-terminal (Figure 3) or centrally positioned within the protein. The 3-D structure of several CBDs in complex with their ligands has been determined which provides insights into the underlying mechanism of CBD-ligand recognition and interaction [44]. The data from these structures hence indicates that CBDs from different families are structurally similar and that their carbohydrate binding capability could be accredited to several aromatic amino acids that comprise the hydrophobic surface. CBDs in hydrolytic proteins like cellulases are linked to CDs via relatively unstructured linker sequence rich in proline and threonine. CBD is thought to function by bringing the biocatalyst into intimate and prolonged association with its recalcitrant substrate thereby increasing the rate of catalysis. They are thus considered important for the initiation and processivity of the enzymes particularly of exoglucanases [20,45,48]. The removal of CBD from enzymatic subunit has found to dramatically decrease the activity of enzymatic subunit. The essential role of CBD has been shown for CBHI, a cellobiohydrolase from *Trichoderma reesei* [49,50]. The CBHI without the CBD i.e., the core enzyme or the catalytic domain was found to have a very limited overall action on cellulose. The catalytic unit was able to initiate the hydrolysis similar to that of the complete enzyme but this activity ceased rapidly and it was concluded that without the activity of binding domain, the hydrolysis would be limited to the readily accessible and/or amorphous regions of the cellulose. Once these regions are hydrolyzed the available substrate sites would deplete, terminating the reaction. However in contrast, in the presence of CBD, the binding sites were incessantly reformed, allowing a continuous activity. CBDs are also thought to direct cellulases to their new target sites, the regions where catalytic action will be most active [34,51,52]. The family 2 CBD once attached was reported to scan the surface of cellulose without dissociation to access new regions susceptible for hydrolysis. In addition CBDs also tend to play a role in sloughing off the cellulose fragments from cellulosic substrates enhancing hydrolysis. They appear to catalyze the disruption of the non-covalent interactions between the cellulose chains allowing erosion of the chains on the surface of the crystal [53,54].

#### Catalytic domain (CD)

The longest domain within cellulases corresponds to catalytic cores. Studies of gene deletion, proteolytic truncation etc., show that they behave as independent entities conferred with catalytic activity and defined specificity towards soluble model substrates [41]. Although the CD of cellulases exhibit considerable diversity, they have been grouped into glycoside hydrolase families based on their amino acid sequence similarities. Glycoside hydrolases are a group of enzymes which hydrolyze the glycosidic bond between carbohydrates or between a carbohydrate and a non-carbohydrate moiety [20,55]. The enzymes in the same family contain similar basic fold based on the idea that a direct relationship exists between amino acid sequence and the folding of the protein. Based on this classification, enzymes having different substrate specificities are sometimes found in the same family; for example, family 5 contains cellulase, xylanases and mannanases, indicating an evolutionary divergence to acquire new specificities. On the other hand, enzymes with same substrate specificity are found in different families; for example, cellulases are found in 11 (5, 6, 7, 8, 9, 12, 44, 45, 48, 61 and 74) different families. This classification which contains more than 5000 glycoside hydrolases are grouped into 130 families. The classification of glycoside hydrolase into structurally determined families provides valuable insights into the structural features of the enzymes, which are more informative than the substrate specificity which was the basis of old IUBMB classification. The 3-D structure of one member of the family can be used to infer the structure of other members of the same family. This classification also defines the domains of the enzymes and thus resolves the contradiction about substrate specificity for multifunctional enzymes [20]. It also sheds light on the evolution of the glycoside hydrolases; as for example, some families are deeply rooted evolutionarily, such as family 9, which contains cellulases of fungi, bacteria, animals and plants. However this is in contrast to family 7 which contains hydrolases of fungal origin only and family 8 which contains only bacterial hydrolases. In addition, cellulases from several families and hence from several different folds are found in the same organism; for example, *Cellulomonas thermocellum* contain endoglucanase and exoglucanase from families 5, 8, 9 and 48. Cellulases are hence a complex group of enzymes that seems to have evolved through concurrence from a repertoire of basic folds. In addition, the extensive diversity within the cellulase families reflects the heterogeneity of cellulose and associated polysaccharide within plant material and a variety of environment where hydrolysis takes place. Proteolytic truncation of CBDs led to the determination of the 3D structures of a variety of CDs by X-ray crystallography. The CD of *Trichoderma reesei* cellobiohydrolase II (CBH II), belonging to glycosyl hydrolase family 6, is a large α/β protein with 5 α-helices and 7 β-strands. The active site is an enclosed tunnel located at the *C*-terminal end of a β-barrel through which the cellulose chain threads [56]. This structure was confirmed in endoglucanase II from *Thermomonospora fusca*, another family 6 member [57]. The 3D structures of catalytic cores of both these enzymes are very similar. However, closer examination reveals a major difference in the degree of active site accessibility to the substrate corresponding to the major difference in their endo- and exo- mode of action [58]. In endoglucanase, the substrate tend to lie in an open cleft which can accommodate cellulose anywhere along the chain, whereas exoglucanase forms an enclosed tunnel in order to allow cellulose chains to be threaded releasing cellobiose or cellooligosaccharides. The hydrolysis of β 1,4-glycosidic bond proceeds via general acid–base catalysis requiring a proton donor and a nucleophile or a base [59,60]. The hydrolysis product can either result in overall retention or inversion of the configuration of the carbon at anomeric position. The stereochemical course of hydrolysis has been determined for several glucanases belonging to different families [61-66]. For example, members of the family 5, 7 and 11 proceeds via retention of the anomeric configuration, whereas enzymes of family 6 and 9 proceeds via inversion mechanism. Generally the overall 3D structure and stereospecificity of hydrolysis are conserved within the family.

### Cellulose degradation mechanisms

There are at least four different known strategies for cellulose degradation as described below.

#### Non-Complexed cellulolysis

**Figure 4:**
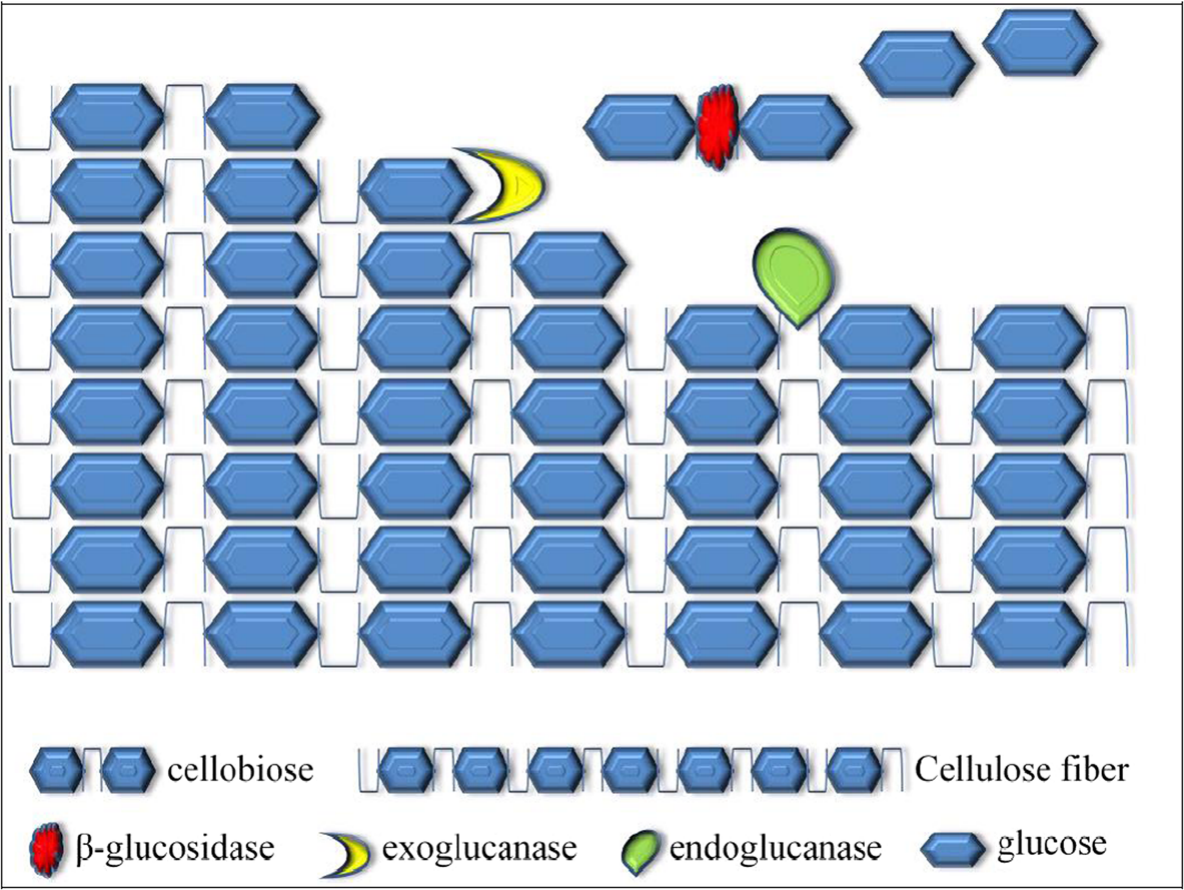
**Hydrolysis of cellulose by non-complexed cellulase system.** The enzymes are secreted extracellularly resulting in the release of sugars which are eventually taken up by the cells and metabolized.

**Figure 5:**
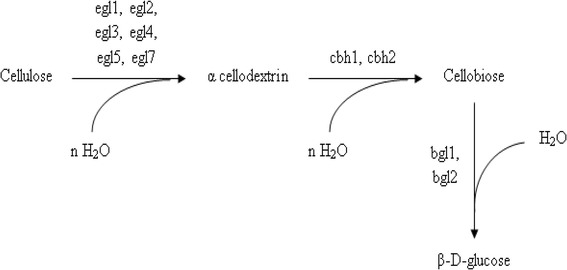
**Cellulose degradation by** ***Trichoderma reesei*** **.** Cellulose is attacked by endoglucanases releasing cellodextrins which is further broken down by exoglucanases into cellobiose. The hydrolysis of cellobiose is then mediated by β – glucosidases releasing glucose.

**Figure 6:**
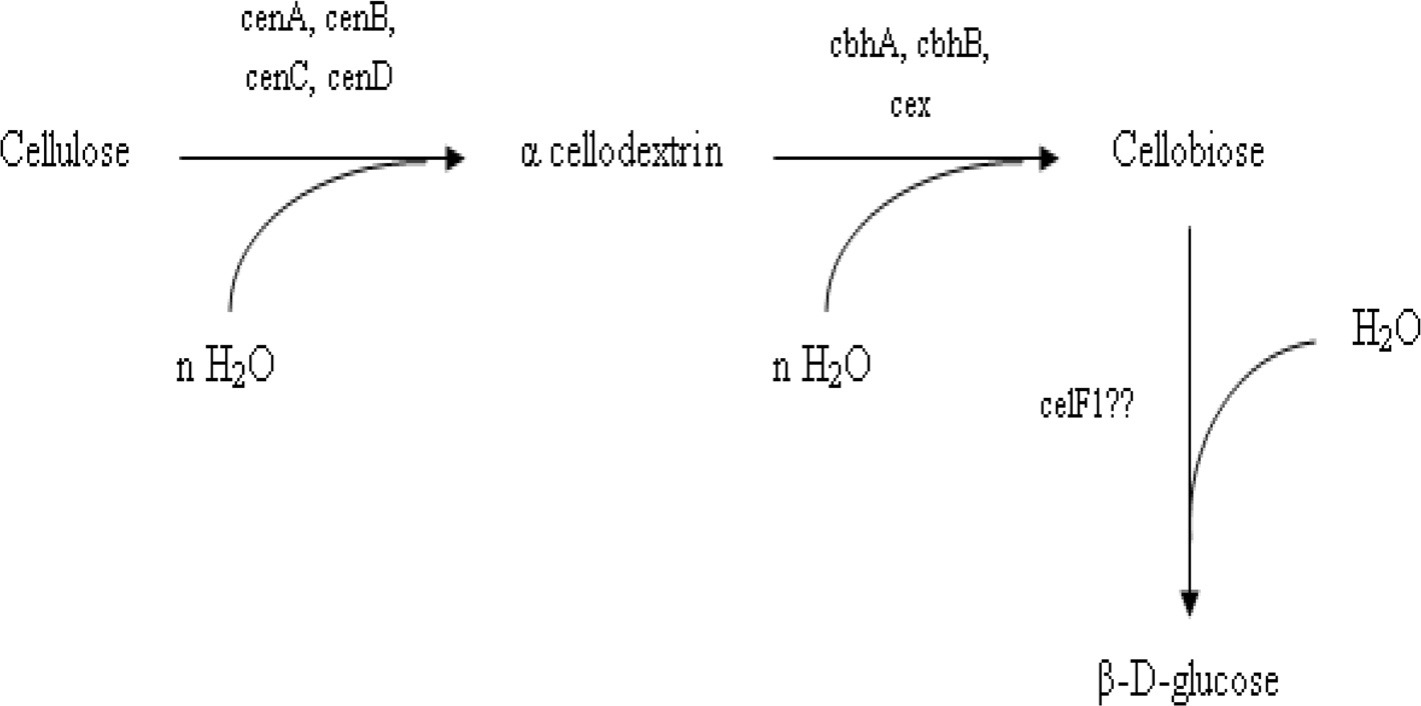
**Cellulose degradation by** ***Cellulomonas fimi*** **.** Cellulose is attacked by endoglucanases releasing cellodextrins which is further broken down by exoglucanases into cellobiose. The hydrolysis of cellobiose is then mediated by β – glucosidases releasing glucose.

In this system, a set of six to ten individual cellulases (with or without CBDs) are produced. They do not form stable complexes. It is mostly observed in aerobic organisms that secrete soluble extracellular cellulolytic enzymes [38,67]. The enzymes attack cellulose, resulting in the release of sugars which are eventually taken up by the cells and metabolized (Figure 4). The best studied non-complexed cellulase systems are those of aerobic fungi such as *Trichoderma reesei* [45,68-70]. *T. reesei* cellulases system consists of five endoglucanases (EGI, EGII, EGIII, EGIV and EGV), two exoglucanases (CBHI and CBHII) and two β-glucosidases (BGLI and BGLII) [71]. Despite the fact that endoglucanases are the main components responsible for decreasing the degree of polymerization of cellulose, they only represent less than 20% of the total cellulase activity of the system [20]. They cleave the cellulose chain internally at relatively amorphous regions creating new chain ends susceptible to the action of cellobiohydrolases, the principal component of the system. The cellobiohydrolases, CBHI and CBHII, constitute about 60 and 20% of the total cellulase activity respectively [72]. The 3D structure of CBHI as determined by X-ray crystallography, shows the presence of 4 surface loops, 50 Å in length giving rise to a tunnel, whereas CBHII has been shown to have two surface loops of 20 Å [56,73]. These tunnels are essential for the processive nature of these enzymes and helps in cleavage of cellulose chain from reducing (CBHI) and non-reducing (CBHII) ends [74-76]. Cellobiose is the major end product of cellobiohydrolase activity, the hydrolysis of which is facilitated by the two β-glucosidases which in turn help to elevate the pressure of feedback inhibition (Figure 5). It is noteworthy that BGLI and BGLII both have been isolated from culture supernatants of *T. reesei*, however a large fraction of these enzymes remain bound to the surface of the cell [77,78]. This may help to limit the loss of degradation product to the competing microbes present in the surrounding environment. The cellulase system of thermophilic fungus, *Humicola insolens* is homologous to that of *T. reesei* system with minor differences. The system consists of two cellobiohydrolases, CBHI & CBHII and five endoglucanases including EGI, EGII, EGIII, EGV & EGVI [79]. Among aerobic bacteria, the best studied cellulolytic species are from the genera *Cellulomonas* and *Thermobifida. Cellulomonas* are Gram positive, non-spore-forming, facultative anaerobic actinomycete that belongs to the Coryneform group of bacteria [80,81]. They produce at least four endoglucanases (CenA, CenB, CenC and CenD), two exoglucanases (CbhA and CbhB) and an exoglucanse with a xylanase activity (Cex) [82-84]. The system resembles those of aerobic fungi (Figure 6). Similarly, a major soil cellulose degrader *T. fusca*, is a thermophilic filamentous bacterium containing 6 cellulases including three endoglucanases (E1, E2 and E5), two exoglucanases (E3 and E6) and an unusual cellulase (E4) with both exo-/endo- activity [20,85]. The ability of cellulolytic filamentous fungi (and actinomycete bacteria) to penetrate cellulosic materials through hyphae enables them to release cellulases in confined cavities within cellulosic materials. These free cellulases therefore suffice for the efficient hydrolysis of cellulose under such conditions [86]. In contrast anaerobic bacteria lack the ability of effective penetration of cellulosic substrates and thus have to find an alternative approach for cellulose degradation in order to gain access (in the presence of other competing microorganisms) to the products of cellulose hydrolysis with the limited ATP available for the synthesis of cellulases. This, in part, could have resulted in the development of ‘complexed cellulase system’ which positions the anaerobic organism close to the site of hydrolysis.

#### Complexed cellulolysis

**Figure 7:**
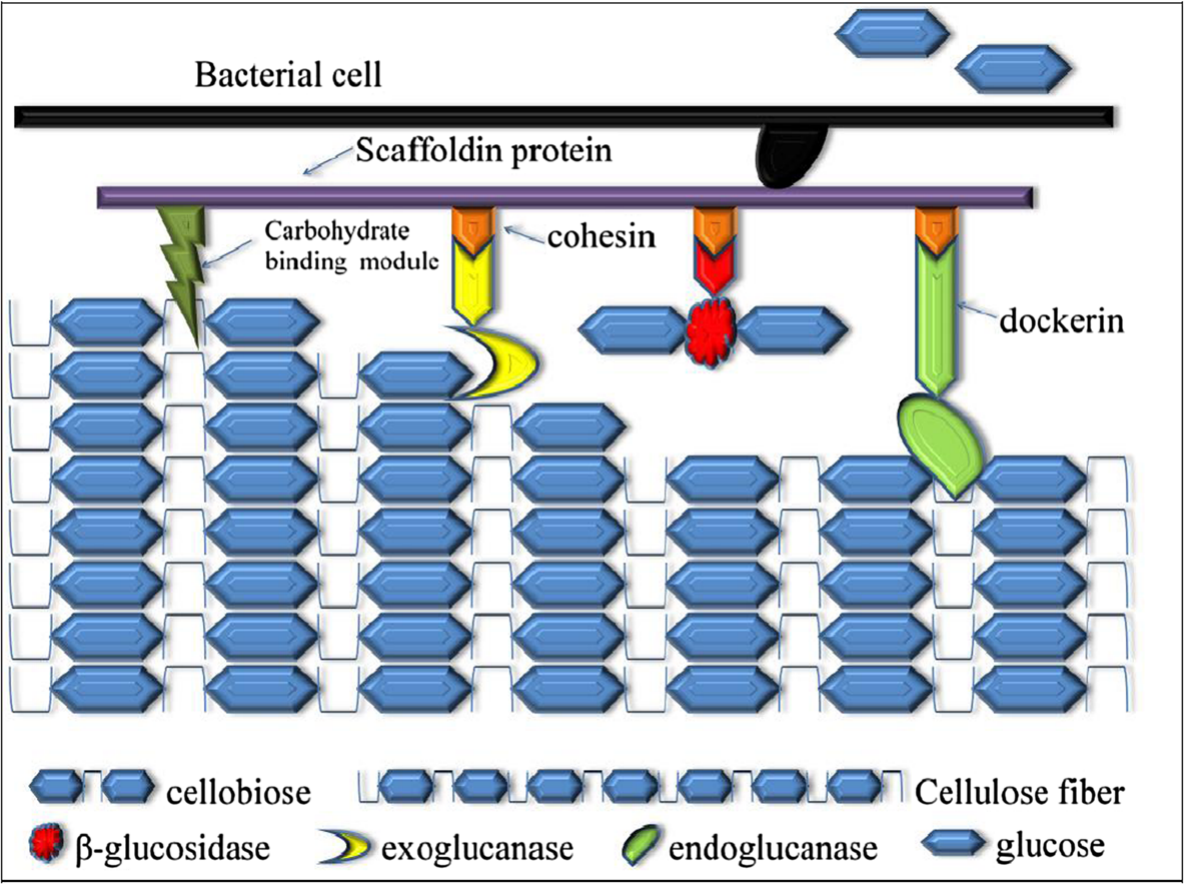
**Hydrolysis of cellulose by complexed cellulase system.** The cellulase components are associated tightly forming multiprotein complexes called “cellulosome” and are found attached to bacterial cells. Components of hydrolysis are released in the vicinity of cells which are immediately taken up by the cells and metabolized.

**Figure 8:**
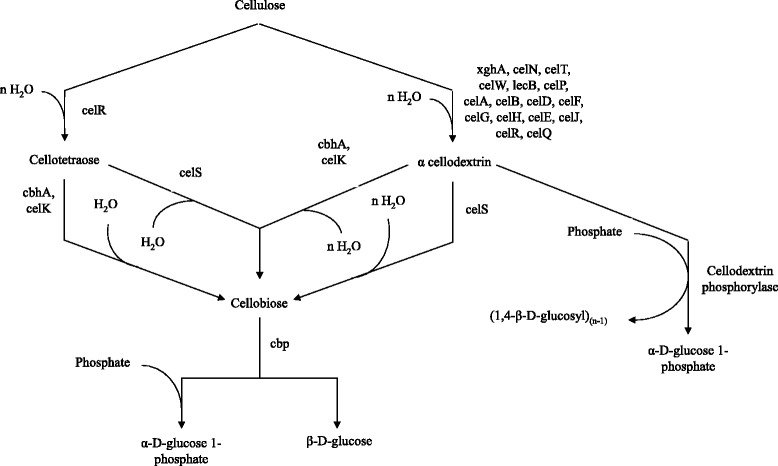
**Cellulose degradation by** ***Clostridium thermocellum*** **.** Cellulose is attacked by various endoglucanases present in cellulosome releasing cellotetraose and/or cellodextrins. These cellooligosaccharides are then attacked by exoglucanases within cellulosome releasing cellobiose which is eventually converted to glucose by β – glucosidase.

The complexed cellulase system is mostly utilized by anaerobic microorganisms and consists of large protein complexes called cellulosome usually attached to the surface of the organism [20,41,87,88] (Figure 7). The term was first coined by Lamed and coworkers in 1983 [89] while studying the cellulase system of *Clostridium thermocellum*. Cellulosome help anchor bacteria to cellulose resulting in localized release of hydrolysis products which are taken up by the cells. Cellulosomes contain numerous kinds of cellulases and related enzyme subunits which are held together by a unique scaffoldin subunits. Scaffoldins are very large, modular polypeptides that hold the multimolecular complex together. They contain a CBD, one or more conserved hydrophilic modules (the function of which is not known) and most importantly multiple copies of cohesin domains. The cellulosomal enzyme subunits are known to contain a dockerin domain, which mediates the integration of enzymes into the cellulosome complex. Dockerins of the enzymatic subunits are involved in a very stable type of binding interaction with the cohesins of the scaffoldin subunit. There is little or no specificity in the binding of various cohesins and the dockerins in the cellulosomes. The CBD helps in the recognition and binding of the scaffoldin subunit to the cellulosic substrate, hence, if the cellulosome is implanted in the cell surface, the CBD of scaffoldin results in the binding of the entire cell to its insoluble substrate, cellulose [42]. Cellulosomes vary in size from 2 – 16 MDa, however in case of polycellulosomes, size can extend up to 100 MDa [19,90]. The scaffolding subunit of cellulosome is sometimes heavily glycosylated with carbohydrate content varying from 6 – 13% and protects the cellulosome from proteolytic enzymes while playing a role in cohesion-dockerin recognition [91]. Under electron microscope cellulosome appears like a fist that opens to spread the catalytic domains allowing it to attach to its substrate [20,42]. Hence these complexes are stable enough to remain bound to bacterial cell while flexible enough to tightly bind to cellulose. Among cellulolytic anaerobes the best characterized example of cellulosome is that of *Clostridium thermocellum*, a Gram positive, sporogenic, strictly anaerobic thermophilic bacterium. The cellulosome structure of *C. thermocellum* has been studied via a combination of various biochemical, structural and genetic analysis [90,92]. The scaffoldin, CipA, of *C. thermocellum* is 197 kDa multimodular protein containing nine cohesins, four hydrophilic modules (X-modules) and a CBD belonging to family III. The genome encodes for at least 9 endoglucanases (CelA, CelB, CelD, CelE, CelF, CelG, CelH, CelN, CelP), 4 exoglucanases (CbhA, CelK, CelO, CelS), 5 hemicellulases (XynA, XynB, XynV, XynY, XynZ), 1 chitinase (ManA) and 1 lichenase (LicB) that have dockerin moiety and can associate with CipA’s cohesion moieties to form a cellulosome. It is noteworthy that cellulosomal components do not only include cellulases but hemicellulases as well, the enzymes responsible for the breakdown of other polymers associated with cellulose in the natural environment. The probable pathway for cellulose degradation by this organism is shown in Figure 8. The exact composition of cellulosome and the assembly of CD on scaffoldin vary with the extracellular environment and the presence of particular polysaccharides. However CelS, a major processive exocellulase with preference for crystalline as well as amorphous cellulose is always present [93]. Evidence suggests that cellulolysis in rumen bacteria and fungi also proceeds via cellulosomes. *Ruminococcus flavefaciens*, a ruminal bacterium when grown in the presence of cellulosic material like cellobiose, a protuberance on the cell surface with production of 1.5 MDa cellulosome like structure is observed which suggests the production of cellulosome [94]. In addition, the isolation of 250 kDa protein from *Ruminococcus albus*, another rumin bacterium indicates the presence of a large scaffoldin [95]. Anaerobic cellulolytic fungi are only found in the rumen of herbivorous animals where they actively produce cellulases. Escalating evidence shows that anaerobic fungi also employ cellulosomal machinery for the hydrolysis of cellulose. The isolation of high molecular weight complexes with binding affinity for microcrystalline cellulose from *Piromyces* sp. strain E2 in addition to the presence of conserved non-catalytic repeat sequence, probably serving a docking function, have also been identified in *Priomyces* as well as *Neocallimastix* species [96,97]. Cellulolysis via cellulosome is generally a highly efficient process and may have a number of advantages including:

- Presence of correct ratio between components of cellulosomal machinery optimizes synergism.
- Optimum spacing and organization of individual components maximizes synergy by avoiding non-productive binding.
- Competition between different components is limited due to the binding of the whole complex to a single site.
- The presence of enzymes with different specificity in one complex avoids the halt in the process on depletion of one structural type of cellulose.

#### Cellulolysis without processive cellulases

The third strategy appears to be utilized by at least two very efficient cellulose degraders: *Cytophaga hutchinsonii*, an aerobic soil cellulolytic organism and *Fibrobacter succinogenes*, an anaerobic ruminal bacterium [34,35,88]. These organisms do not seem to encode for any processive cellulases. Up until recently, the presence of processive cellulase was considered mandatory for effective degradation of crystalline cellulose. Examination of *C. hutchinsonii* genome sequence reveals the presence of clusters of genes responsible for degradation of cellulose [38]. In addition to genes encoding probable β endoglucanases, there are genes encoding possible β glucosidases but no recognizable exoglucanases/cellobiohydrolases. In addition, most cellulases of *C. hutchinsonii* do not contain CBD indicating the absence of any processive cellulases [34,98]. As described above, cellulases generally consist of a CD, a CBD and the linker, joining the two [53,99]. Since to date these features were considered mandatory for the efficient degradation of cellulose by other cellulose degraders, *C. hutchinsonii* may therefore have a novel mechanism for cellulose degradation. The genome sequence of the major cellulolytic rumen bacterium *F. succinogenes* has been determined [34]. It does not seem to possess any processive cellulases nor does it seem to encode for dockerin domains or a scaffoldin gene, as does other anaerobes [100,101]. Furthermore, none of the cellulases from these organisms have much activity on crystalline cellulose [35,38,102]. However both these organisms grow very rapidly on cellulose. They have a unique requirement of physical association with their carbon source and are only able to grow when tightly bound to cellulose. In addition, none of these organisms secrete free cellulases or produce cellulosomes. Hence there appears to be a novel strategy for cellulose utilization by these organisms. One possible mechanism for cellulose digestion by these bacteria might be the one used by starch degrading bacterium, *Bacteroides thetaiotaomicron* [34]. In this model, cellulose is bound to a protein complex present in the outer membrane and the individual molecules are transported into the periplasmic space, where they are degraded by cellulases. In this model, individual cellulose molecules would be readily degraded by endoglucanases and hence excludes the requirement for processive cellulases. This hypothesis also fulfills the unique requirement of these bacteria to be in direct contact with their insoluble substrate for efficient digestion [34,35,38].

#### Cellulolysis via non-hydrolytic enzymes

Lastly, some non-hydrolytic enzymes i.e., oxidative enzymes have also been reported to be associated with cellulose degradation [103]. This system is most thoroughly studied in white-rot fungi, however some cellulolytic bacteria have also been discovered which tend to degrade cellulose via oxidative enzyme strategy [97,103,104]. In addition to hydrolytic enzymes, occasional non-hydrolytic enzymes have also been cited in the literature to be involved in cellulose degradation [103,104]. These include oxidative enzymes such as cellobiose:quinone oxidoreductase (CBQ), cellobiose dehydrogenase and cellobiose oxidase (CBO) [103,105-107]. These enzymes oxidize cellobiose and/or higher cellodextrin reducing ends to their corresponding lactones which can then be used by organisms as their source of carbon [97,104,108]. After the discovery of oxidative enzymes as potential candidate involved in cellulose degradation [103], research has mostly been focused on enzymes from white rot fungi. To date, there is little knowledge about oxidative systems in bacteria [97,104]. The possible role played by these oxidizing enzymes in cellulose degradation may be to alleviate the inhibitory effect of cellobiose on cellobiohydrolase action, to regulate the synthesis of enzymes involved in cellulose degradation and/or in the metabolism of cellobiose itself [97]. In addition since these enzymes are most commonly found in wood rotting fungi, it is suggested that oxidases might also generate H_2_O_2_ required by lignin-degrading enzymes like peroxidases and laccases for the degradation of lignin in natural environment.

### Synergy and competition among cellulose degrading system

The cellulase degradation system has been shown to have a higher collective activity than the sum of the activities of individual enzymes, a phenomenon called synergy [40,46]. Four forms of synergism have been reported:

- Synergism between endoglucanase and exoglucanase, called endo-exo synergy
- Synergism between exoglucanases, called exo-exo synergy
- Synergism between exoglucanase and β – glucosidase to remove cellobiose that inhibits exoglucanase
- Synergism between catalytic and carbohydrate binding domains

Fujita et al., [109] reported the synergistic hydrolysis of amorphous cellulose by a yeast strain co-displaying endoglucanase II (EGII) and cellobiohydrolase II (CBHII) from *Trichoderma reesei* and *Aspergillus aculeatus* β-glucosidase 1 (BGL1). They observed higher hydrolytic activity by the strain co-displaying EGII and CBHII (1.3 mM reducing sugar were released in 60 hours) on amorphous cellulose than the strain displaying only EGII (0.5 mM reducing sugars were released in 60 hours) with the main hydrolysis product been cellobiose. The co-display of BGL1 along with EGII and CBHII resulted in direct production of ethanol from amorphous cellulose. Ethanol was not produced from amorphous cellulose in the presence of only EGII and CBHII. Zhou and Ingram [110] observed synergism between two endoglucanases CelY and CelZ from *Erwinia chrysanthemi*. They observed about 1.8 fold synergy when the enzymes were used in combination. The synergy was due to the difference in substrate preference. CelY hydrolyzed CMC to fragments averaging 10.7 glucosyl units but was unable to hydrolyze cellotetraose and cellopentaose. On the other hand CelZ readily hydrolyzed soluble cellooligosaccharides and amorphous cellulose to produce cellobiose and cellotriose as major end product. Hydrolysis of CMC by CelZ resulted in fragments averaging 3.6 glucosyl units. In combination both enzymes hydrolyzed CMC to fragments averaging 2.3 glucosyl units. Synergy was also observed after the sequential addition of CelY and CelZ (after heat inactivation of CelY) showing that synergy does not require the simultaneous presence of both the enzymes. However no synergy was observed when CelZ was used as the first enzyme, hence showing that only CelY can act independently to modify the substrate to make it more accessible to CelZ. By conventional definition, exocellulases releases cellobiose from non-reducing ends of cellulose chain, which does not explain exo-exo synergy and would hence compete for limited number of hydrolysis sites, instead of cooperating to give synergistic hydrolysis. Enzymatic hydrolysis would be insufficient if the enzymes only act at non-reducing ends, since half the chain ends would be unused. Hence this traditional model was questioned followed by the report describing two exoglucanases from *Aspergillus aculeatus*, one of which attacks from reducing end and the other from non-reducing end of cellulose chain. Similarly two different classes of cellobiohydrolases in *Trichoderma reesei*, CBHI (makes up 60% of total cellulolyticproteins) acting from reducing end of the chain and CBHII (makes up 20% of total cellulolytic proteins) acting from non-reducing end can achieve complete solubilisation, although slow, of cellulose without the help of endoglucanases [40]. Hence these data suggests that the observed exo-exo synergy could be due to the interaction between non-reducing end attacking and reducing end attacking exocellulases.

Most cellulases are composed of CD and CBD that function independently but act synergistically in the disruption and hydrolysis of cellulose fibres. The CBD makes the substrate more accessible to hydrolytic domain by bringing the catalytic module in close proximity to boost hydrolysis [20,45,48]. It is also considered to play a role in sloughing off the cellulose fragments from the surface of cellulose by splitting of the cross linkages. It is also worth mentioning that cellulose degraders always seem to produce multiple enzymes of each class. As an example *Trichoderma reesei* cellulase system consists of two exoglucanases (CBHI and CBHII), five endoglucanases (EGI, EGII, EGIII, EGIV and EGV) and two β-glucosidases (BGLI and BGLII) [71]. *Humicola insolens* produces at least seven cellulases including two cellobiohydrolases (CBHI and CBHII) and five endoglucanases (EGI, EGII, EGIII, EGV and EGVI) [79]. *Thermobifida fusca* contains three endoglucanases (E1, E2 and E5), two exoglucanases (E3 and E6) and a cellulase with both endo-exo activity (E4) [20,85]. The secretion of multiple cellulases of same class could be due to the heterogenous nature of their substrate. As cellulose structure varies from being purely crystalline to purely amorphous with all degrees of order in between, hence some of these enzymes are more effective towards one form of cellulose while others are more effective towards other forms. In addition, it also indicates that although each individual β 1,4-glucosidic bond is chemically identical, the complex nature of the substrate and the environment in which they are present shows that they are not in identical context. Hence therefore one might expect to observe synergy between cellulases of same class as well as of different classes. The combination of these enzymes thus acts synergistically to hydrolyse cellulose. In addition to cellulase these organisms may also possess enzymes to degrade other polysaccharides associated with cellulose such as hemicelluloses, presumably because their breakdown is required to gain access to cellulose fibres [38].

With regard to synergy between cellulases, synergy is observed in some cases but not in other. Anderson et al., [111] reported synergistic effect by a mixture of cel45A, cel6A (an endoglucanse and an exoglucanase respectively from *Humicola insolens*) and β-glucosidase from *Penicillium brasilianum* on amorphous cellulose. However on crystalline cellulose, these enzymes seem to rather inhibit each other owing to the competition for binding sites on cellulose. Whereas some other studies [112,113] with different enzymes showed synergistic effect on crystalline cellulose but not on amorphous cellulose.

### Cellulolytic protists

Most cellulolytic protists are anaerobes, found in the rumen responsible for the degradation of plant material [36]. Cellulolytic flagellated protists are the major cellulose degraders present in the hind gut of lower termites. In addition, the hind gut of wood eating cockroaches harbor many symbiotic protists responsible for the digestion of cellulose rich diets [36,114,115]. The protists tend to endocytose cellulose particles into their food vacuoles whereby, cellulases produced by protists degrade cellulose [116]. However cellulases from protists have hardly been characterized at molecular level due to the difficulty in culturing the anaerobic gut protist community. So far only a limited number of species have been cultured axenically although culture independent techniques have led some advancement in identifying and characterizing cellulases from gut protists [117,118]. Using PCR, fifteen full-length cDNA clones were isolated and sequenced from the protist community of termite, *Reticulitermes sepratus*. They were found to be identical to family 45 cellulases and were originated from hypermastigote protists. Unlike other bacterial and fungal cellulases, the cellulolytic enzymes from protist consist of catalytic domain only [119]. Ancillary domains like cellulose or CBD were not present in these enzymes. The ingested cellulosic material is crunched and ground by the host increasing the surface of cellulose [120]. The cellulosic material is then selectively endocytosed into the food vacuole where it is degraded further by cellulases [119,121]. As mentioned earlier, the role of CBD is to anchor the enzyme to its substrate thereby increasing the concentration of enzyme at the surface of the polysaccharide. High enzyme concentration around the substrate in the food vacuole together with increased surface area of the ground and crunched cellulose may compensate for the lack of CBD in protist cellulases.

*Coptotermes formasanus*, a wood-feeding termite harbours three symbiotic parabasalian flagelates including *Spirotrichonympha leidyi*, *Holomastigotoides mirabile* and *Pseudotrichonympha grassii* [119,122]. These flagellates play an essential role in the digestion of cellulose rich diet. *P. grassii* is however found to utilize high molecular weight cellulose particles whereas; *H. mirabile* and *S. leidyi* tend to degrade low molecular weight cellooligosaccharides [119,123]. Nakashima et al., [122] isolated a novel cellulase gene, *PgCBH-homos* from the flagellated protist *P. grassii*. On the basis of amino acid sequence, PgCBH-homos is found to be similar to GHF 7 (Glycosyl hydrolase family 7) members that mainly consists of fungal cellulases. However clones similar to GHF7 have been detected in *Dictyostelium discoideum*, the social amoebae of the cellular slime mold (*Dictyostelium discoideum* cDNA Project at [124]. On the basis of their tertiary structures and the catalytic mode of action, GHF 7 members are subdivided into two groups: endoglucanases and cellobiohydrolases. PgCBH-homo however has shown to consist of regions similar to Cel7A from *Trichoderma reesei*, a processive cellobiohydrolase forming a tunnel shaped active site [122]. Hence it was predicted to have similar 3D structure to Cel7A and probably functions as a processive cellulase active against crystalline cellulose. The enzyme was also found to contain signal sequence at *N*-terminal corresponding to the fact that the enzymes could be secreted into the food vacuole after endocytosis of wood particles. Another cellulase gene coding for an endoglucanase CFP-EGI, from symbiotic protist *Spirotrichonympha leidyi* present in the hind gut of lower termite *Coptotermes formasanus* was cloned, expressed and characterized [119]. It’s a 33.6 kDa protein and shows sequence similarity with members of GHF 5, a large and growing family of glycosyl hydrolases comprising of endoglucanases, xylanases, mannanases and 1,3-exoglucanases of both aerobic and anaerobic origins. In addition hydrolases of GHF 5 are very diverse and belongs to both prokaryotes and eukaryotes including bacteria, fungi, nematodes, protists and insects. The heterologous CFP-EGI was found to have an optimum pH of 5.8-6.0 and an optimum temperature of 70°C. In 2010, Todoka et. al., heterologously expressed and characterized a cellulase, RsSymEG, from a symbiotic protist of the lower termite, *Reticulitermes speratus*. The amino acid sequence of RsSymEG indicates that it is a GHF 7 cellulase. The lack of insertion sequence responsible for the formation of a tunnel shaped active site conserved among GHF7 CBHs, indicate that RsSymEG is an endoglucanase similar to that of Cel7B, an endoglucanase from *Trichoderma reesei*. However unlike Cel7B, no CBD was detected in RsSymEG and the optimum temperature and pH was found to be 45°C and 6.5 respectively. In addition chromatographic analysis revealed that the preferred substrate for RsSymEG is cellodextrin which is broken down into cellobiose and glucose. Using PCR, 11 different cellulases from symbiotic protists of four different termites were cloned in *Saccharomyces cerevisiae* [125]. The cellulases belong to 3 different glycosyl hydrolase families i.e., GHF 5, 7 & 45 and were found to be more efficient than EG I, a major endoglucanase from *Trichoderma reesei*, in degrading carboxymethyl cellulose. The presence of cellulases from a variety of different glycosyl hydrolase family corresponds to the fact that various cellulases act in synergism to bring efficient hydrolysis of polymeric cellulose [116]. Unlike anaerobic rumen protists that degrade cellulose, *Dictyostelium discoideum*, the amoebae of the cellular slime mold under harsh conditions transforms into a microcyst, containing cellulose rich cell wall [126-129]. Their cell wall mainly consists of four layers: 1, 2a, 2b and 3. Middle layers i.e., layer 2a and 2b are mainly composed of cellulose fibrils and layer 3 i.e., the inner most layer is at least made up of two components: cellulose and proteins [129,130]. As mentioned earlier the cellulose in their cell wall serves as the structural component and help survive hostile conditions. The inability of *Dictyostelium* mutants to synthesize cellulose results in the failure to form viable spores [33]. The spores remain dormant until the conditions are favourable which then stimulates spore germination. The germination is characterized by the activation of spore, budding of the cell wall with swelling of the entire spore and finally, lysis of the cell wall with simultaneous emergence of an amoeba [131]. Cellulolytic activity in *D. discoideum*, first observed by Rosness [127], plays a significant role in the germination process by causing lysis of the cell wall, hence allowing the emergence of the viable amoeba. Rosness observed the degradation of acid swollen cellulose to glucose by extracts from *D. discoideum* at the sorocarp stage of development. The cellulase activity was found to increase rapidly during aging of sorocarp and therefore the cellulases are thought to play a central role in the germination process of this organism. Latter Jones et al. [132], partially characterized two cellulolytic enzymes in this organism released during swelling stage of germination. During this stage the layers 1, 2a and 2b are ruptured and this hence suggests that cellulases during this stage act to degrade layers 2a and 2b celluloses and eventually diffuse into the extracellular medium after the rupture of the cell wall.

## Conclusions

With the completion of the *Acanthamoeba* genome, enzymes for the synthesis and breakdown of cellulose have been identified in *A. castellanii* which are likely to participate in the morphogenesis in this group of organism [133,134]. Based on sequence data, cellulases of *A. castellanii* appear to be similar to bacterial proteins and belong to the glycosyl hydrolase family 5, while that of *D. discoideum* belong to glycosyl hydrolase family 9. Cellulases of GH family 5 are mainly endoglucanase which randomly cut cellulose chain in the middle releasing short chain oligosaccharides like cellodextrins. However, a complete identification, characterization, and localization of enzymes in *Acanthamoeba* with β-glucanase activity, which are likely necessary in morphogenetic events as well as controlled hydrolysis of the cyst wall remain unclear. Based on the known enzymes involved in various cellulose degradation systems, future studies should be carried out to specifically address the following question.

*What is the specific structure of cellulose in Acanthamoeba? how are cellulose fibers in Acanthamoeba organized, ranging from crystalline to amorphous forms? and what is the specific combination of cellulases that can disrupt Acanthamoeba cyst wall integrity.* These are important questions as in patient tissues, *Acanthamoeba* form cysts and are resistant to chemotherapy, leading to recurrence of infection after treatment. It is envisaged that the disruption of cyst wall will make amoeba (concealed within) susceptible to available chemotherapeutic agents. ‘Associative’ therapy should therefore act in conjunction and thus may augment the potency of other compounds, resulting in the improved treatment with reduced infection recurrence. Also cellulose is a structural component limited to some bacteria, protists and higher plants. Consequently, a specific cellulose degrader (i.e., cellulase) should ideally have no and/or minimal effect on non-target (human) cells. Studies should therefore focus on testing the use of these molecules/compounds *in vivo* which may aid in the improved therapy against *Acanthamoeba* infections. These are important questions as in patient tissues, *Acanthamoeba* form cysts and are resistant to chemotherapy, leading to recurrence of infection after treatment. In addition, a thorough understanding of cellulose degradation mechanisms and/or identification of components that can interfere with this process would undoubtedly be of potential value in therapy.
